# Data supporting the high-accuracy haplotype imputation using unphased genotype data as the references

**DOI:** 10.1016/j.dib.2016.06.029

**Published:** 2016-06-29

**Authors:** Wenzhi Li, Wei Xu, Shaohua He, Li Ma, Qing Song

**Affiliations:** a Center of Big Data and Bioinformatics, First Affiliated Hospital of Medicine School, Xi’an Jiaotong University, Xi’an, Shaanxi, China; b Cardiovascular Research Institute, Morehouse School of Medicine, Atlanta, GA, USA; c 4DGenome Inc., Atlanta, GA, USA

## Abstract

The data presented in this article is related to the research article entitled “High-accuracy haplotype imputation using unphased genotype data as the references” which reports the unphased genotype data can be used as reference for haplotyping imputation [1]. This article reports different implementation generation pipeline, the results of performance comparison between different implementations (A, B, and C) and between HiFi and three major imputation software tools. Our data showed that the performances of these three implementations are similar on accuracy, in which the accuracy of implementation-B is slightly but consistently higher than A and C. HiFi performed better on haplotype imputation accuracy and three other software performed slightly better on genotype imputation accuracy. These data may provide a strategy for choosing optimal phasing pipeline and software for different studies.

**Specifications Table**

**Table:** 

Subject area	Biology
More specific subject area	Bioinformatics
Type of data	Tables
How data was acquired	Genotype and haplotype data were obtained from the International HapMap Project database
Data format	Analyzed
Experimental factors	The original data were reformatted to fit the requirement of different software
Experimental features	We generated different implementations from HapMap data set. Then: [1] We compared the performance of different implementations [2]. We compared the phasing performances among HiFi, MACH 1.0, IMPUTE2, BEAGLE.
Data source location	Atlanta, Georgia, USA
Data accessibility	The data are with this article

**Value of the data**

• This data is beneficial to researchers who are interested in haplotyping The data may provide guidance on how to choose the optimal phasing pipeline.
• This data is beneficial to researchers who are interested in imputations and comparison between HiFi and three major phasing software tools (MACH, Impute2 and Beagle) on the accuracy and speed. The data may provide guidance on how to choose the suitable software for different study.
• This data is helpful to compare between HiFi and three major phasing software tools (MACH, Impute2 and Beagle) on their tolerance on statistical reference panels.

## 1 Data

Data presented are summaries of comparison of HiFi performances with three different implementations A, B and C; comparison of HiFi and three standard imputation software performances with molecular reference and statistical reference. The data showed that implementation-B is slightly but consistently higher than A and C; and the data also showed that HiFi performed better on haplotype imputation accuracy and speed,three other tools performed slightly better on genotype imputation.

## 2 Experimental design, materials and methods

### 2.1 Acquisition and processing of HapMap data for different implementations

We downloaded CEU (CEPH, U.S. Utah residents with ancestry from northern and western Europe) chromosome 1 genotype data and haplotype data from HapMap in text format [5], [6]. We use the original haplotype data as molecular reference. To generate the statistical haplotype reference panel, we erased the phase information from those trio haplotypes downloaded from HapMap, and then used the software Beagle version 3.3.2 to resolve the haplotypes from the unphased genotypes. Then we generated following three different implementations by Beagle version 3.3.2: (A) Beagle statistical phasing of unrelated persons and Mendelian-inheritance-based phasing of trios, and then pools the results together; (B) Beagle statistical phasing of pooled unrelated persons and trios, but presumes all as unrelated; and (C) Beagle statistical phasing of pooled unrelated persons and trios, and specifying the family structure in the input. And we chose same 6 samples [2] for further analysis.

### 2.2 Comparison of HiFi performances with three different implementations A, B and C

We compared the HiFi performances with three different implementations. Our data showed that the performances of these three implementations are similar on accuracy, in which the accuracy of implementation-B is slightly but consistently higher than A and C (Table S1).

### 2.3 Comparison of HiFi and three standard imputation software performances with molecular reference and statistical reference

We compared the performance between HiFi and three standard imputation software tools (MACH, IMPUTE2 and BEAGLE) [7], [8], [9]. As the result, HiFi performed better on haplotype imputation accuracy (Table S2) and speed (Table S4), whereas MACH, IMPUTE2 and BEAGLE performed slightly better on genotype imputation accuracy (Table S3), in which MACH and IMPUTE2 performed the best on genotype imputation.
